# MortalityPredictors.org: a manually-curated database of published biomarkers of human all-cause mortality

**DOI:** 10.18632/aging.101280

**Published:** 2017-08-31

**Authors:** Maximus V. Peto, Carlos De la Guardia, Ksenia Winslow, Andrew Ho, Kristen Fortney, Eric Morgen

**Affiliations:** ^1^ BioAge Labs, Berkeley, CA 94703, USA

**Keywords:** database, mortality, aging, biomarker, population-based

## Abstract

Biomarkers of all-cause mortality are of tremendous clinical and research interest. Because of the long potential duration of prospective human lifespan studies, such biomarkers can play a key role in quantifying human aging and quickly evaluating any potential therapies. Decades of research into mortality biomarkers have resulted in numerous associations documented across hundreds of publications. Here, we present MortalityPredictors.org, a manually-curated, publicly accessible database, housing published, statistically-significant relationships between biomarkers and all-cause mortality in population-based or generally healthy samples. To gather the information for this database, we searched PubMed for appropriate research papers and then manually curated relevant data from each paper. We manually curated 1,576 biomarker associations, involving 471 distinct biomarkers. Biomarkers ranged in type from hematologic (red blood cell distribution width) to molecular (DNA methylation changes) to physical (grip strength). Via the web interface, the resulting data can be easily browsed, searched, and downloaded for further analysis. MortalityPredictors.org provides comprehensive results on published biomarkers of human all-cause mortality that can be used to compare biomarkers, facilitate meta-analysis, assist with the experimental design of aging studies, and serve as a central resource for analysis. We hope that it will facilitate future research into human mortality and aging.

## INTRODUCTION

Mortality biomarkers are of great clinical and research interest. General clinical applications include identifying high-risk patient groups, prognosticating for individual patients, and helping healthcare providers decide among treatment options [1]. Examples of very well-studied mortality biomarkers include blood pressure, cholesterol, and waist circumference, which have well-established relationships with mortality in various populations documented in dozens of studies, some with thousands or millions of participants [2-4]. These traditional biomarkers have been joined in more recent years by many biomarkers utilizing modern assays, for example genome-wide methylation levels [5], cell-free DNA concentration [6], and leukocyte telomere length [7].

Biomarkers of human mortality are also centrally important to research on human aging, due largely to the long potential duration of prospective studies on human lifespan. This can be a tremendous obstacle both in terms of resources (i.e. money to support such lengthy trials) and delayed progress (i.e. each research result could take decades to obtain). Mortality biomarkers have solved similar problems in the past by providing surrogate endpoints for crucial clinical outcomes, facilitating studies that might otherwise have been prohibitively expensive or time consuming [8, 9]. Blood pressure and cholesterol are two of many markers that have played this role in the past, by facilitating cardiovascular research aimed at reducing morbidity and mortality [10]. Such biomarkers have also gained clinical importance as surrogate markers in clinical practice, where treatments are often initiated with the explicit goal of changing a patient's biomarker value [11-13]. While this approach has important potential drawbacks [8, 10], it is certainly more practical for a patient to track how a new intervention affects her blood pressure or serum cholesterol, rather than how it affects her lifespan, which is unknown until death.

Abundant research on mortality biomarkers has resulted in numerous associations documented across hundreds of publications, generating an unwieldy collection of data that can be difficult for researchers or clinicians to interpret or use effectively. There have been no recent attempts to collate this data nor, to our knowledge, to provide tools for locating, organizing, or comparing data from relevant studies. In the present article, we describe an effort to facilitate a more comprehensive and effective approach to evaluating the literature in this area. We present MortalityPredictors.org, a manually-curated, publicly accessible database housing published, statistically-significant relationships in humans between biomarkers and all-cause mortality in population-based or generally healthy samples. To our knowledge, this is the first publicly available resource to collect such information, and we hope it will encourage: 1) the allocation of resources to mortality biomarkers with the greatest potential for accurately predicting human all-cause mortality, 2) efforts to construct multi-biomarker models to further improve such accuracy, and 3) research on human aging and therapies that aim to slow aging or otherwise reduce mortality.

## RESULTS

### Data description

**Figure 1:**
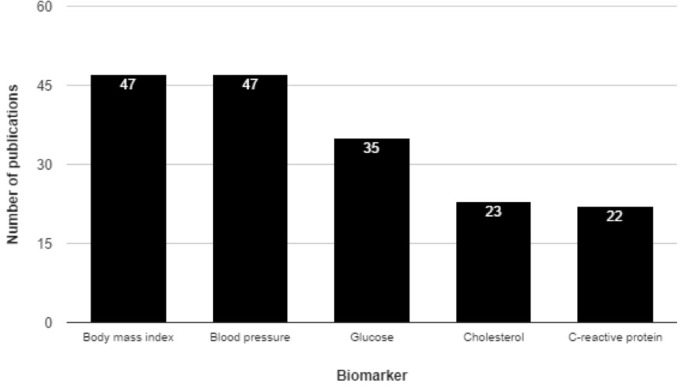
Top five most commonly studied human biomarkers of all-cause mortality by number of publications The top five most commonly studied biomarkers in the database are shown here. The bar height indicates the number of publications associated with each, and this number is explicitly shown in white near the top of each bar.

The initial PubMed query yielded 3,841 publications; these were narrowed to 833 after screening the publication abstracts. Further articles were excluded based on details from the full-text article, and data was finally extracted from the remaining 589 articles, yielding 1,576 reported associations involving 471 unique biomarkers. Figure 1 illustrates the 5 most commonly studied biomarkers in our data set, which encompasses a diverse array of biomarker types, including both blood and urinary biomarkers, numerous physical parameters (BMI, strength, skinfold thickness, etc.), and other features including electrocardiography, spirometry, protein levels, epigenetic features, medical imaging, and others. These types are shown in Table 1, along with descriptive information. The largest number of biomarkers and publications were seen within the “blood” type (i.e. biomarkers assayed via sampling of peripheral blood) (Table 2).

**Table 1:** Biomarker types according to the number of curated biomarkers

Biomarkers	Publications	Type	Example biomarkers	Largest normalized effect size
165	310	Blood	Apolipoprotein A-1, Mean corpuscular volume, Lymphocyte percentage	8.33
30	35	Composite	Body mass index and leg extensor strength, Lipid accumulation product, Hypothyroidism	7.93
10	14	Computed tomography	Bone density, Thigh intramuscular fat, Skeletal muscle density	12.28
4	4	Echocardiography	Interventricular septum thickness, Left ventricular ejection fraction, Left ventricular hypertrophy	6
61	45	Electrocardiography	Low frequency power of heart rate variability, QRS Transition, counterclockwise rotation, QTc dispersion minimum value	14.29
79	3	Epigenetics	cg14575484, cg16197857, cg27635330	2.45
10	25	Exercise test	Exercise capacity, Strength, Cardiorespiratory fitness	6.67
22	24	Other	Relative abdominal fat, Basal metabolic rate, Interday rhythm stability	8.4
47	94	Physical parameter	Arm circumference, Lean mass index, Body mass index	16.9
10	58	Sphygmomanometry	Blood pressure, Pulse pressure, Mean arterial pressure	4.2
11	14	Spirometry	Peak expiratory flow, Forced expiratory volume, Forced vital capacity	4.59
13	12	Ultrasonography	QUI stiffness, Bone mineral density, Broadband ultrasound attenuation	4.89
12	23	Urine	Creatinine excretion, Proteinuria, Sodium (24-hour excreted)	11

**Table 2:** Top 10 biomarkers by number of publications, within the blood biomarker type

Name	Publications	Results	Largest normalized effect size	Best p value
Glucose	35	43	4.1	0.0001
Cholesterol	23	29	5.08	0.001
C-reactive protein	22	26	3.64	0.0001
25-hydroxyvitamin D	21	24	4.93	0.0001
Estimated glomerular filtration rate	15	22	7.8	0.0001
Uric acid	12	15	2.8	0.001
High-density lipoprotein cholesterol	12	12	2.38	0.0002
White blood cell count	12	31	3.33	9.22E-31
Glycated hemoglobin	11	14	3.2	0.001
Testosterone	11	20	2.3	0.001

### Browsing the database

**Figure 2:**
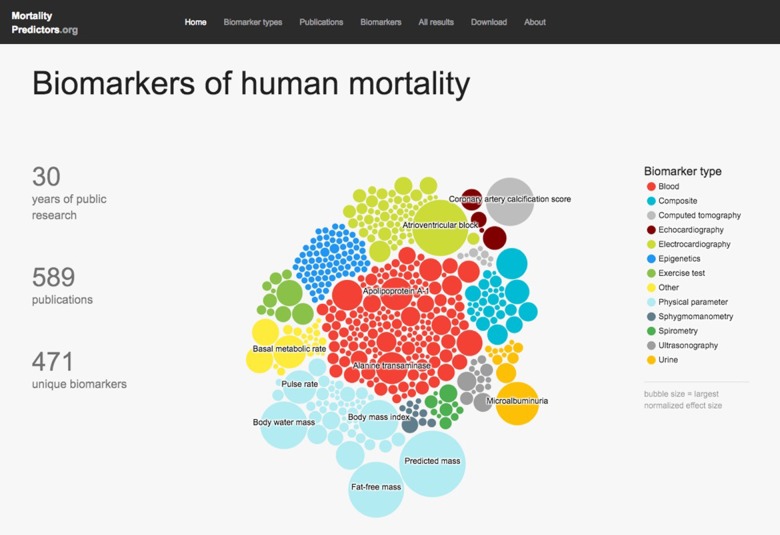
MortalityPredictors.org Database homepage This is a screen capture of the database home page. Major database statistics are summarized on the left. In the center, an interactive bubble diagram displays a colored bubble for each database biomarker. Each bubble's color corresponds to the biomarker type (color key shown at right), and the size corresponds to the largest normalized effect size for that marker. Clicking on a bubble leads to the database page for that biomarker. At the top, the main database section labels are shown as hyperlinks that lead to those portions of the database.

The home page for the database (Figure 2) displays summary information about the data and lists four main sections in the database: 1) biomarker types, 2) publications, 3) biomarkers, and 4) all results. These sections present, respectively, all the main types of biomarkers included in the database, all source publications for included data, all studied biomarkers, and all curated associations. The entries in each section can be sorted or filtered by relevant parameters, such as number of publications, publication year, or effect size. Excessively long data fields are truncated, which is signaled by “…” at the end; the full field can be viewed by hovering the mouse over the field of interest.

### Searching the database

Each main section has its own search field that can be used to filter entries based on the primary data field for that section. For instance, one can find a particular biomarker by typing its name into the search field in the “Biomarkers” section.

### Downloading the database

Users can easily download the raw data comprising the entire database via a “Download all data” link in the “Downloads” section.

### Example use-cases

#### Finding the biomarker with the strongest published association to all-cause mortality

**Figure 3:**
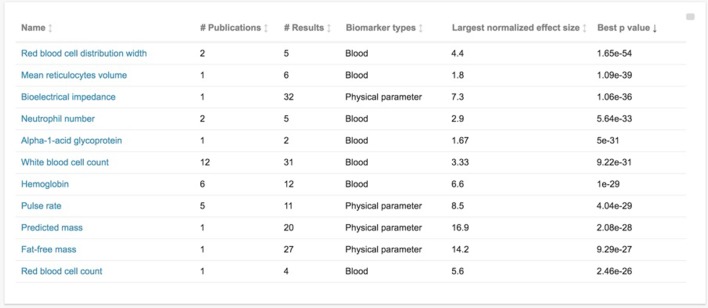
Database biomarkers sorted by statistical significance of their reported associations This is a screen capture of the database “Biomarkers” section, sorted by “best p-value” for that biomarker in increasing order. “Best p-value” refers to the lowest p-value among curated studies for that biomarker. Other presented information on each biomarker includes the number of curated publications, the number of curated associations resulting from those publications (“# Results”), the biomarker type, and the largest normalized effect size for that biomarker (as defined in the manuscript text).

This is accomplished by navigating to the Biomarkers section and sorting by “Best p value” (by clicking the heading for best p value). This shows that “red blood cell distribution width” has the smallest p value at 1.65e-54 (see Figure 3). Clicking on the name of the biomarker then leads to the individual page for that marker, including basic background information about the marker, as well as entries for each curated association. We can see that p=1.65e-54 was for an association with a normalized hazard ratio (HR) of 4.4, and that the extremely low *p*-value is mostly likely explained by the extremely large sample size, at more than 227,000 male participants from in the UK Biobank cohort. The analogous association for the 271,000 female participants from this cohort is easily found on the same page, with HR=3.3, p=4.79e-24 for the same comparison.

#### Finding the peripheral blood biomarker with the largest published effect size

This is accomplished by navigating to the “Biomarker types” section, selecting “Blood”, and sorting the resulting page by “Largest normalized effect size” in descending order (by clicking this heading twice). This reveals that the largest change in risk of all-cause mortality was recorded for Apolipoprotein A-1, which is a major component of high-density lipoprotein (HDL) cholesterol and helps to clear fat from white blood cells and arterial plaques of atherosclerosis. Clicking on the biomarker name “Apolipoprotein A-1” allows the user to further explore its associations, revealing that the study with the largest effect size had a normalized hazard ratio of 8.33 per 1 unit biomarker increase (p=0.029) and was based on a sample of 331 people from a northwest London population sample with a mean follow-up of 24 years.

## DISCUSSION

MortalityPredictors.org is a unique resource encompassing 1,587 entries from over 30 years of published research, and catalogs a diverse array of biomarkers, reports details of their relationships with all-cause mortality, and includes relevant meta-data. While certain biomarker databases already exist, for example the Infectious Disease Biomarker Database (IDBD; http://biomarker.cdc.go.kr; [14]) and the Early Detection Research Network Biomarker Database (EDRNBD; http://edrn.nci.nih.gov/biomarkers), these are limited to specialized applications or specific diseases and do not share the generalizability of our mandate, which emphasizes a) all-cause mortality and b) human studies in population-based or relatively disease-free cohorts. Some research groups have performed systematic reviews and/or meta-analyses on biomarkers of all-cause mortality, but these have been either within specific domains [15], or limited to very specific time-frames or purposes [1], and have not made their results available as a public database. Other groups have evaluated large panels of candidate biomarkers within specific cohorts, for example 106 metabolites in 10 thousand participants in the Estonian Biobank (Fischer, 2015), but these represent individual studies with little attempt to summarize or collate the work of others. Finally, a number of aging-related databases exist as well, including Human Aging Genomic Resources (HAGR; http://genomics.senescence.info/; [16]), GeroProtectors database (http://geroprotectors.org/; [17]), and the JenAge Aging Factor Database (AgeFactDB; http://agefactdb.jenage.de/; [18]), but none focus on mortality biomarkers and the curation of reported associations.

### Applications of MortalityPredictors.org

This publicly-available dataset at Mortality Predictors.org provides an overview of the current state of research on human biomarkers of all-cause mortality, and is of great potential utility for both research and clinical applications. We envision a number of applications that employ its meta-data and search capabilities. These might include:

#### Identifying optimal biomarkers to assess a therapy's impact on mortality

Researchers designing studies to measure the potential mortality benefit of a therapy may want to begin by testing its effect on various biomarkers of mortality. If a therapy causes positive changes in those mortality biomarkers, then it might warrant the additional expense and effort of a long-term study to directly assess its true effect on mortality. Such researchers would be able to use MortalityPredictors.org initially to choose which biomarkers to assess in their study sample, and might be interested in selecting those with the most extreme effect sizes that have been reported in the largest number of studies.

#### Identifying optimal mortality biomarkers of a specific type (e.g. blood, urine)

Researchers interested in mortality biomarkers might have constraints on the type of biomarkers that are useful to them. A retrospective patient cohort might have blood banked on all participants, but no capability for other follow-up testing, so the ability to easily find information on biomarkers of the “blood” type in our database could facilitate experimental design. Alternatively, a prospective cohort in a remote or impoverished area with a limited budget or limited access to technology might be much more amenable to physical parameters that are simple and inexpensive to measure, such as body mass index and blood pressure, rather than molecular or protein-based markers requiring more elaborate testing infrastructure. Such researchers could use MortalityPredictors.org to specifically find biomarkers of the type most appropriate for their study design.

#### Choosing optimal biomarkers for monitoring overall health

Researchers or clinicians trying to construct an optimal panel of biomarkers for measuring general health -- such as in the context of a broad-purpose cohort study or preventive clinical care -- might wish to go beyond the tests traditionally used to screen for common diseases at recommended ages, and construct a panel of easily measured biomarkers directly associated with mortality. In a clinical context, results from such tests would have to be interpreted and communicated to patients with caution, given the paucity of well-validated research on many biomarkers. Nonetheless, MortalityPredictors.org should be uniquely helpful in designing such a panel.

#### Performing a systematic review or meta-analysis of mortality biomarkers

Researchers interested in performing a systematic review and/or meta-analysis on any particular mortality biomarker or group thereof could to use the database to ascertain useful parameters to plan their research (such as how many studies are available in the area, the range of test modalities, effect sizes, *p*-values, etc), to quickly identify a large number of studies on their topic of interest, or to supplement their own independent search strategy with additional articles.

### The potential of multi-predictor models

There is great potential for leveraging mortality biomarkers beyond the scope of strategies discussed so far. Importantly, multiple biomarkers can be combined to create indexes that more accurately estimate individual mortality risk than any individual marker [4, 19]. Many such indexes have been created for the prediction of disease risk or of mortality within very specific clinical contexts, and are currently in clinical use. These include the Framingham Risk Score for cardiovascular disease risk [20], the Reynolds Risk Score for cardiac event or stroke risk [21], and the QKidney score for assessment of kidney disease risk [22], among others [23]. The creation of similar indexes, or other sophisticated multi-variable predictors for all-cause mortality will be an important strategy for applying the predictive capability of mortality bio-markers in research and clinical applications. We anticipate that MortalityPredictors.org, by summarizing the known effects of individual markers, will help provide the foundation upon which future work to investigate multi-predictor models can be based.

### Importance of mortality biomarkers for quantifying age-related phenotypes

Population demographics are changing, with dramatically more people over the age of 65 in many countries, a trend which threatens to place ever greater strain on healthcare systems [24]. However, the great burden of this phenomenon does not come from increasing age alone, but more specifically from the development of frailty, an age-related state of increased vulnerability to external stressors and accelerated decline in health [25]. Frailty may occur in individuals at different chronological ages and with different severities, and is caused by the gradual accumulation of age-related damage at the molecular and cellular level, becoming manifest when such damage exceeds the physiological capacity to compensate [26]. Frailty is thus closely related to the concept of “biological age” [27, 28] and one of its most relevant and well-defined implications is an increased risk of death [25, 29]. Consequently, improving biomarkers and multi-predictor models for all-cause mortality may lead to better, more biologically- and clinically-relevant models of both frailty and of biological age, with potential for use in development of interventions that can ameliorate or reverse age-related physiological decline.

One concrete way that such positive impact may occur is via the use of mortality biomarkers as surrogate outcomes for research and clinical intervention, an application of biomarkers mentioned above [8, 9]. To the extent that better biomarkers and predictive models for mortality are identified, this will improve their ability to act as surrogate endpoints for studies that would otherwise not have been performed, or would have required much greater time and expense. At present, there are multiple drugs that have shown promise in model organisms to positively affect the aging process itself, including metformin, acarbose, angiotensin receptor blockers (ARBs) [30], and rapamycin [31, 32], while other therapies, such as senescent cell removal, are now also starting to move towards clinical trials [33]. It is hoped that such treatments can directly influence the molecular and cellular determinants of biological age in order to decrease frailty, thereby preventing age-related disease and disability while extending both lifespans and healthspans. However, the clinical trials that will be necessary to establish the presence or absence of benefit from these therapies may be surprisingly difficult. In addition to standard regulatory and ethical challenges, the scale of human lifespan is such that while results of mouse lifespan experiments are available within a few years, human lifespan or healthspan trials would take decades. This becomes starkly prohibitive in terms of time and expense to evaluate each promising therapy, a situation that can only be improved with reliable biomarkers of mortality and frailty. With biomarkers (or combinations thereof) being used as clinical endpoints, the duration and expense of such trials can be reduced dramatically.

## CONCLUSIONS

Mortality biomarkers have played a major role in facilitating healthcare and research for many decades, and promise to have an ever greater role in the near future, given an aging population in many countries, as well as the rise of frailty and age-related deterioration as health as major public health concerns and active targets for new therapies. Studies evaluating therapies that aim to address the roots of frailty within the aging process itself will benefit greatly from research to identify reliable predictors of mortality. These include the landmark Targeting Aging with Metformin (TAME) trial, now underway, as well as future research into promising therapies such as rapamycin and senescent cell removal. The creation of better predictive models for mortality begins with comprehensive, public data about prior biomarker research, toward which effort we contribute MortalityPredictors.org. Our future research will leverage high-throughput “-omics” technologies to screen large numbers of predictors and derive their combinations that most accurately predict mortality. These will be combined with the best predictors from prior research in order to build and validate powerful multi-predictor models that we hope will accelerate clinical research efforts to target and reduce mortality in older adults.

## MATERIALS AND METHODS

### Study selection

We identified relevant abstracts by searching the PubMed database (http://www.ncbi.nlm.nih.gov/ pubmed) from inception to November 2015 for abstracts reporting on human biomarkers of all-cause mortality studied in either generally-healthy or population-based human samples. We used the query “(biomarker*[Title/Abstract] OR predict*[Title/Abstract] OR associate*[Title/Abstract]) AND (“all-cause mortality”[Title/Abstract] OR “all cause mortality” [Title/Abstract]) NOT (patient[Title/Abstract] OR patients[Title/Abstract])”.

### Curation strategy

Each abstract was assessed by a single curator to determine whether the paper reported a statistically significant (nominal p-value < 0.05) association between at least one biomarker and all-cause mortality. Publications reporting only on biomarkers associated with a specific cause of death, such as cancer or cardiovascular mortality, were excluded. Studies of patient groups (i.e. patients suffering from one or more diseases) were excluded. To help ensure that data were not represented more than once in our database, reviews and meta-analyses were also excluded. In cases of uncertainty regarding inclusion or exclusion criteria, consensus among two curators was sought.

The full-text of each retained publication was reviewed and information regarding characteristics of the study, population, biomarker, and association, were extracted. The prospectively-defined fields used in the mortalitypredictors.org database included: 1) biomarker name, e.g. “interleukin-6”; 2) type of test measure, e.g. blood or electrocardiogram; 3) association effect size and its type, e.g. “hazard ratio of 1.5”; 4) the associated p-value; 5) biomarker comparison groups, e.g. “value >5 vs ≤2”; 6) sample size that the association was based on; 7) adjustment covariates for multivariate analysis, e.g. age, sex, smoking status; 7) population characteristics, e.g. age range, predominant ethnicity, geographic area; 8) follow-up time in years; and 9) the official cohort or study name, if applicable. An additional field was then created from this data called “normalized effect size”, which contains either the effect size (for effect sizes ≥1) or one divided by the effect size (for effect sizes <1). Given that the direction of the effect size (i.e. above or below 1) is determined somewhat arbitrarily by the selection of which comparison group in a study will be the “baseline” group, the normalized effect size allows for comparison of effect size magnitudes across studies.

### Database construction

The user interface for the database was written primarily in Elm 0.17, which compiles to HTML, CSS, and JavaScript that can be displayed directly in a web browser. The database entries are embedded directly in this interface and thus no separate database server is required. The homepage's interactive data visualization was written with the JavaScript library D3.js 4.4.0. Data cleaning and preparation were done using Python 2.7.12 along with the Jupyter notebook 4.2.1.
